# Supplementary material for: Heat adaptation of phage T7 under an extended genetic code
Source: Virus Evol. 2021 Dec 1;7(2):veab100. doi: 10.1093/ve/veab100 (PMC8923235; doi:10.1093/ve/veab100)
Supplement: veab100_Supp [file veab100_supp.zip › Sequencing_Analysis.zip!/Sequencing_Analysis/A2/evidence/SNP_7.html]

BRESEQ :: Evidence


| Predicted mutation | | | | | | |
| --- | --- | --- | --- | --- | --- | --- |
| evidence | seq id | position | mutation | annotation | gene | description |
| RA | NC\_001604.1 | 24,161 | A→C | intergenic (–/–) | – / – | –/– |

| Read alignment evidence... | | | | | | | | | | | |
| --- | --- | --- | --- | --- | --- | --- | --- | --- | --- | --- | --- |
|  | seq id | position | | ref | new | freq | score (cons/poly) | reads | annotation | genes | product |
| \* | NC\_001604.1 | 24,161 | 0 | A | C | 82.6% | 687.7 / 41.0 | 265 | intergenic (–/–) | *–/–* | –/– |
| Reads supporting (aligned to +/- strand):  **ref** base A (29/17);  **new** base C (111/107);  **total** (141/124) | | | | | | | | | | | |
| Fisher's exact test for biased strand distribution *p*-value = 1.46e-01 | | | | | | | | | | | |
| Kolmogorov-Smirnov test that lower quality scores support variant *p*-value = 9.98e-01 | | | | | | | | | | | |

Only 100 of 269 total aligned reads displayed.  

`GTACTGTTAAGCTGCGTGACTTGGCTCTGGAGCGCGCTCGCCGTGCTAACTTCCAAGCGGACCAGATTATCGCTAAGTACGCAATGGGCCACGGTGGTCTTCGCCCAGAAGCTGCTGGTGCAGTGGTTTTCAAAGTGGAGTAATGCTGGGGGTGGCCTCAACGGTCGCTGCTAGTCCCGAAGAGGCGAGTGTTACTTCAACAGAAGAAACCTTAACGCCAGCACAGGAGGCCGCACGCACCCGCGCTGCTAACAAAGCCCGAAAGGAAGCTGAGTTGGCTGCTGCCACCGCTGAGCAATAACTAGCATAACCCCTTGGGGCCTCTAAACGGGTCTTGA‑GGGGTTTTTTGCTGAAAGGAGGAACTATATGCGCTCATACGATATGAACGTTGAGACTGCCGCTGAGTTATCAGCTGTGAACGACATTCTGGCGTCTATCGGTGAACCTCCGGTATCAACGCTGGAAGGTGACGCTAACGCAGATGCAGCGAACGCTCGGCGTATTCTCAACAAGATTAACCGACAGATTCAATCTCGTGGATGGACGTTCAACATTGAGGAAGGCATAACGCTACTACCTGATGTTTACTCCAACCTGATT  >  NC_001604.1/23862‑24461`  

                                                                                                                                                                                                                                                                                                           `|`                                                                                                                                                                                                                                                                                                               
`gtaCTGTTAAGCTGCGTGACTTGGCTCTGGAGCGCGCTCGCCGTGCTAACTTCCAAGCGGACCAGATTATCGCTAAGTACGCAATGGGCCACGGTGGTCTTCGCACAGAAGCTGCTGGTGCAGTGGTTTTCAAAGTGGAGTAATGCTGGGGGTGGCCTCAACGGTCGCTGCTAGTCCCGAAGAGGCGAGTGTTACTTCAACAGAAGAAACCTTAACGCCAGCACAGGAGGCCGAACGCACCCACGCTGCTAACAAAGCCCGAAAGGAAGCTGAGTTGGCTGCTGCCACCGCTGAGCAATaa                                                                                                                                                                                                                                                                                                              >  1:791600/1‑301 (MQ=255)`  
   `cTGTTAAGCTGCGTGACTTGGCTCTGGAGCGCGCTCGCCGTGCTAACTTCCAAGCGGACCAGATTATCGCTAAGTACGCAATTGGCCACGGTGGTCTTCGCCCAGAAGCTGCTGGTGCAGTGGTTTTCAAAGTGGAGTAATGCTGGGGGTGGCCTCAACGGTCGCTGCTAGTCCCGAAGAGGCGAGTGTTACTTCAACAGAAGAAACCTTAACGCCAGCACAGGAGGCCGCACGCACCCGCGCTGCTAACAAAGCCCGAAAGGAAGCTGAGTTGGCTGCTGCCACCGCTGAGCAATCACTa                                                                                                                                                                                                                                                                                                           >  1:187619/1‑301 (MQ=255)`  
     `gTTAAGCTGCGTGACTTGGCTCTGGATCGCGCTCTCCGTGCTAACTTCCAAGCGGACCAGATTATCGCTAAGTACGCAATGGGCCACGGTGGTCTTCGCCCAGAAGCTGCTGGTGCAGTGGTTTTCAAAGTGGAGTAATGCTGGGGGTGGCCTCAACGGTCGCTGCTAGTCCCGAAGAGGCGAGTGTTACTTCAACAGAAGAAACCTTAACGCCAGCACAGGAGGCCGCACGCACCCGCGCTGCTAACAAAGCCCGAAAGGAAGCTGAGTTGGCTGCTGCCACCGCTGAGCAATCACTAGc                                                                                                                                                                                                                                                                                                         <  1:124074/301‑1 (MQ=255)`  
          `gCTGCGTGACTTGGCTCTGGAGCGCGCTCGCCGTGCTAACTTCCAAGCGGACCAGATTATCGCTAAGTACGCAATGGGCCACGGTGGTCTTCGCCCAGAAGCTGCTGGTGCAGTGGTTTTCAAAGTGGAGTAATGCTGGGGGTGGCCTCAACGGTCGCTGCTAGTCCCGAAGAGGCGAGTGTTACTTCAACAGAAGAAACCTTAACGCCAGCACAGGAGGCCGCACGCCCCCGCGCTGCTAACAAAGCCCGAAAGGAAGCTGAGTTGGCTGCTTCCACCGCTGAGCAATCACTAGCATAAc                                                                                                                                                                                                                                                                                                    >  1:218193/1‑301 (MQ=255)`  
             `gCGTGACTTGGATCTGGAGCGCGCTCGCCGTGCTAACTTCCAAGCGGACCAGAGTATCGCTAAGTACGCAATGGGCCACGGTGGTCTTCGCCCAGAAGCTGCTGGTGCAGTGGTTTTCAAAGTGGAGTAATGCTGGGGGTGGCCTCAACGGTCGCTGCTAGTCCCGAAGAAGCGAGTGTTACTTCAACAGAAGAAACCTTAACGCCAGCACAGGAGGCCGCACGCACCCGCGCTGCTAACAAAGCCCAAAAGGAAGCTGAGTTGGCTGCTGCCACCGCTGAGCAATCACTAGCATAAcccc                                                                                                                                                                                                                                                                                                 <  1:16248/301‑1 (MQ=255)`  
                 `aaatgtgtataagagacagccttccggggtAACTTCCAAGCGGACCAGATTATCGCTAAGTACGCAATGGGCCACGGTGGTCTTCGCCCAGAAGCTGCTGGTGCAGTGGTTTTCAAAGTGGAGTAATGCTGGGGGTGGCCTCAACGGTCGCTGCTAGTCCCGAAGAGGCGAGTGTTACTTCAACAGAAGAAACCTTAACGCCAGCACAGGAGGCCGCACGCACCCGCGCTGCTAACAAAGCCCGAAAGGAAGCTGAGTTGGCTGCTGCCACCGCTGAGCAATCACTAGCATAACCGCTTgg                                                                                                                                                                                                                                                                                             <  1:221596/272‑1 (MQ=255)`  
                   `attGGCTCTGGAGCGCGCTCGCCGTGCTAACTTCCAAGCGGACCAGATTATCGCTAAGTACGCAATGGGCCACGGTGGTCTTCGCCCAGAAGCTGCTGGTGCAGTGGTTTGCAAAGTGGAGTAAGGCTGGGGGTTGCCGCAACGGTCGCTGCTAGTCCCGAAGAGGCGAGTGTTACTTCAATAGAAGAAACCTTAACGCCAGCACAGGAGGCCGCACGCACCCGCGCTGCTAACAAAGCCCGAAAGGAAGCTGAGTTGGCTGCTGCCACCGCTGAGCAATCACTAGCATAACCCCTTgggg                                                                                                                                                                                                                                                                                           <  1:824462/300‑1 (MQ=255)`  
                       `gCTCTGGAGCGCGCTCGCCGTGCTAACTTCCAAGCGGACCAGATTATCGCTAAGTACGCAATGGGCCACGGTGGTCTTCGCCCAGAAGCTGCTGGTTCAGTGGTTTTCAAAGTGGAGTAATGCTGGGGGTGGCCTCAACGGTCGCTGCTAGTCCCGAAGAGGCGAGTGTTACTTCAACAGAAGAAACCTTAACGCCAGCACAGGAGGCCGCACGCACCCGCGCTGCTAACAAAGCCCGAAAGGAAGCTGAGTTGGCTGCTGCCACCGCTGAGCAACACCTAGAATAACCCCTTGGGGCctc                                                                                                                                                                                                                                                                                       >  1:762969/1‑301 (MQ=255)`  
                       `gCTCTGGAGCGCGCTCGCCGTGCTAACTTCCAAGCGGACCAGATTATCGCTAAGTACGCAATGGGCCACGGTGGTCTTCGCCCAGAAGCTGCTGGTGCAGTGGTTTTCAAAGTGGAGTAATGCTGGGGGTGGCCTCAACGGTCGCTGCTAGTCCCGAAGAGGCGAGTGTTACTTCAACAGAAGAAACCTTAACGCCAGCACAGGAGGCCGCCCGCACCCGCGCTGCTAAAAAAGCCCGCAAGGAAGCTGAGTTGGCTGCTGCCACCGCTGAGCAATCACTAGCATAACCCCTTGGGGCctc                                                                                                                                                                                                                                                                                       >  1:806309/1‑301 (MQ=255)`  
                          `ctGGAGCTCGCTCGCCGTGCTAACTTCCAAACGGACCAGATTATCGCTAAGTACGCAATGGGCCACGGTGGTCTTCGCCCAGAAGCTGCTGGTGCAGTGGTTTTCAAAGTGGAGTAATGCTGGGGGTGGCCTCAACGGTCGCTGCTAGTCCCGAAGAGGCGAGTGTTACTTCAACAGAAGAAACCTTAACGCCAGCACAGGAGGCCGCACGCACCCGCGCTGCTAACAAAGCCCGAAAGGAAGCTGAGTTGGCTGCTGCCACCGCTGAGCAATAACTAGCATAACCCCTTGGGGCCTATaa                                                                                                                                                                                                                                                                                    >  1:811038/1‑301 (MQ=255)`  
                            `ggAGTGCGCTCGCCGTGCTAACTTCCAAGCGGACCAGATTATCGCTAAGTACGCAATGGGCCACGGTGGTCTTCGCCCAGAAGCTGCTTGTGCAGTGGTTTTCAAAGTGGAGTAATGCTGGGGGTGGCCTCAACGGTCGCTGCTAGTCCCGAAGAGGCGAGTGTTACTTCAACAGAAGAAACCTTAACGCCAGCACAGGAGGCCGCACGCACCCGCGCTGCTAACAAAGCCCGAAAGGAAGCTGAGTTGGCTGCTGCCACCGCTGAGCAATCACTAGCATAACCCCTTGGGGCCTCTAAAc                                                                                                                                                                                                                                                                                  >  1:225670/1‑301 (MQ=255)`  
                               `gcgcgaTCGCCGTGCTAACTTCCAAGCGGACCAGATTATCGCTAAGTACGCAATGGGCCACGGTGGTCTTCGCACAGAAGCTGCTGGTGCAGTGGTTTTCAAAGTGGAGTAATGCTGGGGGTGGCCTCAACGGTCGCTGCTAGTCCCGAAGAGGCGAGTGTTACTTCAACAGAAGAAACCTTAACGCAAGCACAGGAGGCCGCACGCACCCGCGCTGCTAACAAAGCCCGAAAGGAAGCTGAGTTGGCTGCTGCCACCACTGAGCAATCACTAGCATAACCCCTTGGGGCCTCTAAACggg                                                                                                                                                                                                                                                                               <  1:14984/301‑1 (MQ=255)`  
                                      `ggctatGCTAACTTCCAAGCGGCCCGGATTATCGATAAGTACGCAATGGGCCCCGGTGGTCTTCGCCCAGAAGCTGCTGGTGCAGTGGTTTTCAAAGTGGAGTAATGCTGGGAGTGGCCTCAACGGTCGCTGCTAGTCCCGTAGAGGCGAGTGTTACTTCAACAGACGAAACCTTAACGCCAGCACAGGAGGCCGCACGCACACGCGCTGATAACAAAGCCAGAAAGGAAGCTGAGTTGGCTGCTGCCACAGCTGAGCACTCACTAGCATAACCCCTTGGGGCCTCTAAACGGGTCTTGA‑g                                                                                                                                                                                                                                                                       <  1:692284/296‑1 (MQ=255)`  
                                                             `ccAGATTATCGCTAAGTACGCAATGGGCCACGGTGGTCTTCGCCCAGAAGCTGCTGGTGCAGTGGTTTTCAAAGTGGAGTAATGCTGGGGGTGGCCTCAACGGTCGCTGCTAGTCCCGAAGAGGCGAGTGTTACTTCAACAGAAGAAACCTTAACGCCAGCACAGGAGGCCGCACGCACCCGCGCTGCTAACAAAGCCCGAAAGGAAGCTGAGTTGGCTGCTGCCACCGCTGAGCAATCACTAGCATAACCCCTTGGGGCCTCTAAACCTGTCtcttatacacatctccgagcccacgaga                                                                                                                                                                                                                                                 >  1:293220/1‑274 (MQ=255)`  
                                                              `cAGATTATCGCTAAGTACGCAATGGACCACGGTGGTCTTCGCCAAGAAGCTGCTGGTGCAGTGGTTTTCAAAGTGGAGTAATGCTGGGGGTGGCCTCAACGGTCACTGCTAGTCCCGAAGAGGCGAGTGTTACTTCAACAGAAGAAACCTTAACGCCAGCACAGGAGGCCGCACGCACCCGCGCTGCTAACAAAGCCCGAAAGGAAGCTGAGTTGGCTGCTGCCACCGCTGAGCAATCACTAGCATAACCCCTTGGGGCCTCTAAACGGGTCTTGA‑GGGGTTTTTTGCTGAAAGGAGGAAc                                                                                                                                                                                                                                               <  1:727815/301‑1 (MQ=255)`  
                                                                `gATTATCGCTAAGTACGCAATGGGCCACGGTGGTCTTCGCCCAGAAGCTGCTGGTGCAGTGGTTTTCAAAGTGGAGTAATGCTGGGGGTGGCCTCAACGGTCGCTGCTAGTCCCTAAGAGGCGAGTGTTACTTCAACAGAAGAAACCTTAACGCCAGCACAGGAGGCCGCACGCACCCGCGCTGCTAACAAAGCCCGAAAGGAAGCTGAGTTGGCTGCTGCCACCGCTGAGCAATAACTAGCATAACCCCTTGGGGCCTCTAAACGGGTCTTGA‑GGGGTTTTTTGCTGAAAGGAGGAACta                                                                                                                                                                                                                                             >  1:14265/1‑301 (MQ=255)`  
                                                                `gATTATCGCTAAGTACGCAATGGGCCACGGTGGTCTTCGCCCAGAAGCTGCTGGTGCAGTGGTTTTCAAAGTGGAGTAATGCTGGGGGTGGCCTCAACGGTCGCTGCTAGTCCCGAAGAGGCGAGTGTTACTTCAACAGAAGAAACCTTAACGCCAGCACAGGAGGCCGCACGCACCCGCGCTGCTAACAAAGCCCGAAAGGAAGCTGAGTTGGCTGCTGCCACCGCTTAGCAATCACTAGCATAACCCCTTGGTGCCTCTAAACGGGTCTTGA‑GGGGTTTTTTGCTGAAAGGAGGAACta                                                                                                                                                                                                                                             >  1:389057/1‑301 (MQ=255)`  
                                                                        `cTAAGTACGCAATGGGCCACGGTGGTCTTCGCCCAGAAGCTGCTGGTGCAGTGGTTTTCAAAGTGGAGTAATGCTGGTGGTGGCCTCAACGGTCGCTGCTAGTCCCGAAGAGGCGAGTGTTACTTCAACAGAAGAAACCTTAACGCCAGCACAGGAGGCCGCACGCACCTGCGCTGCTAACAAAGCCCGAAAGGAAGCTGAGTTGGCTGCTGCCACCGCTTAGCAATAACTAGCATAACCCCTTGTGGCCTCTAAACGTGTCTTGA‑GGGGTTTTTTGCTGAAAGGAGGAACTATATGCGCt                                                                                                                                                                                                                                     <  1:419525/301‑1 (MQ=255)`  
                                                                            `gTACGCAATGGGCCACGGTGGTCTTCGCCCATAAGCTTCTGGTGCAGTGGTTTTCAAAGTGGAGTAATGCTGGGGGTGGCCTCAACGGTCGCTGCTAGTCCCGAAGAGGCGAGTGTTACTTCAACAGAAGAAACCTTAACACCAGCACAGGAGGCCGCACGAACCCGCGCTGCTAACAAAGCCCGAAAGGAAGCTGAGTTGGCTGCTGCCACCGCTGAGCAATCACTAGCATAACCCCTTGGGGCCTCTAAACGGGTCTTGA‑GGGGTTTTTTGCTGAAAGGAGGAACTATATGCGCTCAtg                                                                                                                                                                                                                                 >  1:547801/1‑300 (MQ=255)`  
                                                                                `gCAATGGGCCACGGTGGTCTTCGCCCAGAAGCTGCTGGTGCAGTGGTTTTCAAAGTGGAGTAATGCTGGGGGTGGCCTCAACGGTCGCTGCTAGTCCCGAAGAGGCGAGTGTTACTTCAACAGAAGAAACCTTAACGCCAGCACAGGAGGACGCACGCACCCGCGCTGCTAACAAAGCCCGAAAGGAAGCTGAGTTGGCTGCTGCCACCGCTGAGCAATCACTAGCATAA‑CCCTTGGGGCCTCTAAACGGGTCTTGA‑GGGGTTTTTTGCTGAAAGGAGGAACTATATGCGCTCATGCAata                                                                                                                                                                                                                            <  1:260644/301‑1 (MQ=255)`  
                                                                                     `gggCCACGGTGGTCTTCGCCCAGAAGCTGCTGGTGCAGTGGTTTTCAAAGTGTAGTAATGCTGGGGGTGGCCTCAACGGTCGCTGCTAGTCCCGAAGAGGCGAGTGTTACTTCAACAGAAGAAACCTTAACGCCAGCACAGGCGGCCGCACGCACCCGCGCTGCTAACAAAGCCCGAAAGGAAGCTGAGTTGGCTGCTGCCACCGCTGAGCAATCACTAGCATAACCCCTTGGGGCCTCTAAACGGGTCTTGA‑GGGGTTTTTTGCTGAAAGGCGGAACTATATGCGCTCATTCGATATGaa                                                                                                                                                                                                                        >  1:818239/1‑301 (MQ=255)`  
                                                                                      `ggCCACGGTTGTCTTCGCCCAGAAGCTGCTGGTGCAGTGGTTTTCAAAGTGGAGTAATGCTGGGGGTGGCCTCAACGGTAGCTGCTAGTCCCGAAGAGGCGAGTGTTACTTCAACAGAAGAAACCTTAACGCCAGCACAGGAGGCCGCACGCACCCGCGCTGCTAACAAAGCCCGAAAGGAAGCTGAGTTGGCTGCTGCCACCGCTGAGCAATCACTAGCATAACCCCTGGGGGCCTCTAAACGGGGCTTGA‑GGGGTTTTTTGCTGAAAGGAGGAACTATATGCGCTCATGCGATATGAAc                                                                                                                                                                                                                       >  1:57605/1‑301 (MQ=255)`  
                                                                                             `gtggtCTTCGCCTAGAAGCTGCTGGTGCAGTGGTTTTCAAAGTGGAGTAATGCTGGGGGTGGCCTCAACGGTCGCTGCTAGTCCCGAAGAGGCGAGTGTTACTTCAACAGAAGAAACCTTAACGCCAGCACAGGAGGCCGCACGCACCCGCGCTGCTAACAAAGCCCGAAAGGAATCTGAGTTGGCTGCTGCCACCGCTGAGCAATCACTAGCATAACCCCTTGGGGCCTCTAAACGTGTCTTGA‑GGGGTTTTTTGCTGAAAGGAGGAACTATATGCGCTCATGCGATATGAACGTTgaga                                                                                                                                                                                                                >  1:136933/1‑301 (MQ=255)`  
                                                                                             `gtggtCTTCGCCCAGAAGCTGCTGGTGCAGTGGTTTTCAAAGTGGAGTAATGCTGGGGGTGGCCTCAACGGTCGCTGCTAGTCCCGAAGAGGCGAGTGTTACTTCAACAGAAGAAACCTTAACGCCAGCACAGGAGGCCGCACGCACCCGCGCTGCTAACAAAGCCCGAAAGGAAGCTGAGTTGGCTGCTGCCACCGCTGAGCAATCACTAGCATAACCCCTTGGGGCCTCTAAACGGGTCTTGA‑GGGGTTTTTTGCTGAAAGGAGGAACTATATGCGCTCATGCGATATGAACGTTgaga                                                                                                                                                                                                                <  1:594501/301‑1 (MQ=255)`  
                                                                                                `gtCTTCGCCCAGAAGCTGCTGGTGCAGTGGTTTTCAAAGTGGAGTAATGCTGGGGGTGGCCTCAACGGTCGCTGCTAGCCCCGAAGAGGCGAGTGTTACTTCAACAGAAGAAACCTTAACGCCAGCACAGGAGGCCGCACGCACCCGCGCTGCTAACAAAGCCCGAAAGGAAGCTGAGTTGGCTGCTGCCACCGCTGAGCAATCACTAGCATAACCCCTTGGGGCCTCTAAACGGGTCTTGA‑GGGGTTTTTTGCTGAAAGGAGGAACTATATGCGCTCATGCGATATGAAcctgtctctta                                                                                                                                                                                                             >  1:544320/1‑291 (MQ=255)`  
                                                                                                      `gCCCAGAGGCTGCTGGTGCAGTGGTTTTCAAAGTGGAGTAATGCTGGTGGTGGCCTCAACGGTCGCTGCTAGTCCCGAAGAGGCTAGTGTTACTTCAATAGAAGAAACCTTAACGCCAGCACATGAGGCCGCACGCACCCGCGCTGCTAACAAAGCCCGAAAGGAAGCTGAGTTGGCTGCTGCCACCGCTGAGCAATCACTAGCATAACCCCTTGGGGCCTCTAAACGGGTCTTGA‑GGGGTTTTTTGCTGAAAGGAGGAACTATATGCGCTCATGCGATATGAACGTTGAGACTGCCGCTg                                                                                                                                                                                                       <  1:570845/301‑1 (MQ=255)`  
                                                                                                         `agagacaggctgGTGCAGTGGTTTTCAAAGTGGAGTAATGCTGGGGGTGGCCTCAACGGTCGCTGCTAGTCCCGAAGAGGCTAGTGTTACTTCAACAGAAGAAACCTTAACGCCAGCACAGGAGGCCGCACGCACCCGCGCTGCTAACAAAGCCCGAAAGGAAGCTGAGTTGGCTGCTGCCACCGCTGAGCAATCACTAGCATAACCCCTTGGGGCCTCTAAACGGGTCTTGA‑GGGGTTTTTTGCTGAAAGGAGGAACTATATGCGCTCATGCGATATGAACGTTGAGACTGCCGCTGAGt                                                                                                                                                                                                    <  1:470733/293‑1 (MQ=255)`  
                                                                                                           `gAAGCTGCTGGTGCAGTGGTTTTCAAAGTGGAGTAATGCTGGGGGTGGCCTCAACGGTCGCTGCTAGTCCCGAAGAGGCGAGTGTTACTTCAACAGAAGAAACCTTAACGCCAGCACAGGAGGCCGCACGCACCCGCGCTGCTAACAAAGCCCGAAAGGAAGCTGAGTTGGCTGCTGCCACCGCTGAGCAATCACTAGCATAACCCCTTGGGGCCTCTAAACGGGTCTTGA‑GGGGTTTTTTGCTGAAAGGAGGAACTATATGCGCTCATGCGATATGAACGTTGAGACTGCCGCTGAGTTa                                                                                                                                                                                                  >  1:164206/1‑301 (MQ=255)`  
                                                                                                                `gacaggggCAGTGGTTTTCAAAGTGGAGTAATGCTGTGGGTGGCCTCAACGGTCGCTGCTAGTCCCGAAGAGGCGAGGGTTACTTCAACAGAAGAAACCTTAACGCCAGCACAGGAGGCCGCACGCACCCGCGCTGCTAACAAAGCCCGAAAGGAAGCTGAGTTGGCTGCTGCCACCGCTGAGCAATCACTAGCATAACCCCTTGGGGCCTCTAAACGTGTCTTGA‑GGGGTTTTTTGCTGAAAGGAGGAACTATATGCGCTCATGCGATATGAACGTTGAGACTGCCGCTGAGTTATCAGc                                                                                                                                                                                             <  1:704712/294‑1 (MQ=255)`  
                                                                                                                `aaaaagagacagggTGTTCAAATTGGAGTAATGCTGGGGGTGGCCTAAACGGTCGCTGCTAGTCCCGACGAGGCGAGTGTTACTTCAACAGAAGAAACCTTAACGCCAGCACAGGAGGCCGCACGCACCCGCGCTGCTAACAAAGCCCGAAAGGAAGCTTAGTTGGCTGCTGCCACCGCTGAGCAATCACTAGCATAACCCCTTGGGGCCTCTAAACGGGTCTTGA‑GGGGTTTTTTGCTGAAAGGAGGAACTATATGCGCTCATGCGATATGAACGTTGAGACTGCCGCTGAGTTATCAGc                                                                                                                                                                                             <  1:738740/289‑1 (MQ=255)`  
                                                                                                                          `gttgTTTTCAAAGTGGAGTAATGCTGGGGGTGGCCTCAACGGTCGCTGCTAGTCCCGAAGAGGCGAGTGTTACTTCAACAGAAGAAACCTTAACGCCAGCACAGGAGGCCGCACGCACCCGCGCTGCTAACAAAGCCCGAAAGGAAGCTGAGTTGGCTGCTGCCACCGCTGAGCAATCACTAGCATAACCCCTTGGGGCCTCTAAACGGGTCTTGA‑GGGGTTTTTTGCTGAAAGGAGGAACTATATGCGCTCATGCGATATGAACGTTGAGACTGCCGCTGAGTTATCAGCTGTGAACGAc                                                                                                                                                                                   >  1:314396/4‑301 (MQ=255)`  
                                                                                                                          `gTGGTTTTCAAAGTGGAGTAATGCTGGGGGTGGCCTCAACGGTCGCTGCTAGTCCCGAAGAGGCGAGTGTTACTTCAACAGAAGAAACCTTAACGCCAGCACAGGAGGCCGCACGCACCCGCGCTGCTAACAAAGCCCGAAAGGAAGCTGAGTTGGCTGCTGCCACCGCTGAGCAATCGCTAGCATAACCCCTTGGGGCCTCTAAACGGGTCTTGA‑GGGGTTTTTTGCTGAAAGGAGGAACTATATGCGCTCATGCGATATGAACGTTGAGAcctgtctcttatacacatctccgagcccc                                                                                                                                                                                   >  1:630610/1‑273 (MQ=255)`  
                                                                                                                          `gTGGTTTTCAAAGTGGAGTAATGCTGGGGGTGGCCTCAACGGTCGCTACTAGTCCCGAAGAGGCGAGTGTTACTTCAACAGAAGAAACCTTAACGCCAGCACAGGAGGCCGCACGCACCCGCGCTGCTAACAAAGCACGAAAGGAAGCTGAGTTGGCTGCTGCCACCGCTGAGCAATAACTAGCATAACCCCTTGGGGCCTCTAAACGGGTCTTGA‑GGGGTTTTTTGCTGAAAGGAGGAACTATATGCGCTCATGCGATATGAACGTTGAGACTGCCGCTGAGTTAACAGCTGTGAACGAc                                                                                                                                                                                   >  1:282616/1‑301 (MQ=255)`  
                                                                                                                               `tttCAAAGTGGAGTAATGCTGGGGGTGGCCTCAACGGTCGCTGCTAGTCCCGAAGAGGCGAGTGTTACTTCAACAGAAGAAACCTTAACGCCAGCACAGGAGGCCGCACGCACCCGCGCTGCTAACAAAGCCCGAAAGGAAGCTGAGTTGGCTGCTGCCACCGCTGAGCAATCACTAGCATAACCCCTTGGGGCCTCTAAACGGGTCTTGA‑GGGGTTTTTTGCTTAAAGGAGGAACTATATGCGCTCATGCGATATGAACGTTGAGACTGCCGCTGAGTTATCAGCTGTGAACGACATTCt                                                                                                                                                                              <  1:532773/301‑1 (MQ=255)`  
                                                                                                                                   `gtataagagacaggCTGGGGGTGGCCTCAACGGTCGCTGCTAGTCCCGAAGAGGCGAGTGTTACTTCAACAGAAGAAACCTTAACGCCAGCACAGGAGGCCGCACGCACCCGCGCTGCTAACAAAGCCCGAAAGGAAGCTGAGTTGGCTGCTGCCACCGCTGAGCAATCACTAGCATAACCCCTTGGGGCCTCTAAACGGGTCTTGA‑GGGGTTTTTTGCTGAAAGGAGGAACTATATGCGCTCATGCGATATTAACGTTGAGACTGCCGCTGAGTTATCAGCTGTGAACGACATTCTGGCg                                                                                                                                                                          <  1:632059/288‑1 (MQ=255)`  
                                                                                                                                   `aaaGTGGAGTAATGCGGGGGGTGGACTCAATGGGTGCTGCTAGTCCCGAAGAGGCGAGTGTTACTTCAACAGAAGAAACCTTAACGCCAGCACAGGAGGCAGCACGCACCCGCGCTGCGAACAAAGCCCGAAAGGAAGCTGAGTTGGCTGCTGCCACCGCTGAGCAATCACTAGCATAACCCCTTGGGGCCTCTAAACGGGTCTTGA‑GGGGTTTTTTGCTGAAAGTAGGAACTAGATGCGCTCATGCGATATGAACGTTGAGACTGCCGCTGAGTTATCAGCTGTGAACGACATTCTGGCg                                                                                                                                                                          <  1:520492/301‑1 (MQ=255)`  
                                                                                                                                    `aaGTGGAGTAATGCTGGGGGTGGCCTCAACGGTCGCTGCTAGTCCCGAAGAGGCGAGTGTTACTTAAACAGAAGAAACCTTAACGCCAGCACAGGAGGCCGCACGCACCCGCGCTGCTAACAAAGCCCGAAAGGAAGCTGAGTTGACTGCTGCCACCGCTGAGCAATCACTAGCATAACCCCTTGGGGCCTCTAAACGGGTCTTGA‑GGGGTTTTTTGCTGAAAGGAGGAACTATATGCGCTCATGCGATATGAACGTTGAGACTGCCGCTGAgctgtctcttatacacatctccgagcccc                                                                                                                                                                         >  1:748872/1‑273 (MQ=255)`  
                                                                                                                                      `gTGGAGTAATGCTGGGGGTGGCCTCAACTGTCGCTGCTAGTCCCGGAGAGGCGAGTGTTACTTCAACAGAAGAAACCTTAACGCCAGCACAGGAGGCCGCACGCACCCGCGCTGCTAACAAAGCCCGAAAGGAAGCTGAGTTGGCTGCTGCCACCGCTGAGCAATAACTAGCATAACCCCTTGGGGCCTCTAAACGGGTCTTGA‑GGGGTTTTTTGCTGAAAGGAGGAACTATATGCGCTCATGCGATATGAACGTTGAGACTTCCGCTGAGTTATCAGCTGTGAACGACATTCTGGCGTCt                                                                                                                                                                       <  1:41187/301‑1 (MQ=255)`  
                                                                                                                                      `gTGGAGTAATGCTGGGGGTGGCCTCAACGGTCGCTGCTAGTCCCGAAGAGGCGAGTGTTACTTCAACAGAAGAAACCTTAACGCCAGCACAGGAGGCCGCACGCACCCGCGCTGCTAACAAAGCCCGAAAGGAAGCTGAGTTGGCTGATGCCACCGCTGAGCAATCACTAGCATAACCCCTTGGGGCCTCTAAACGGGTCTTGA‑GGGGTTTTTTGCTGAAAGGAGGAACTATATGCGCTCATGCGATATGAACGTTGAGACTGCCGCTGAGTTATCAGCTGTGAACGACATTCTGGCGttt                                                                                                                                                                       >  1:480264/1‑299 (MQ=255)`  
                                                                                                                                        `ggAGTAATGCTGGGGGTGGCCTCAACGGTCGCTGCTAGTCCCGAAGAGGCGAGTGTTACTTCAACAGAAGAAACCTTAACTCCAGCACAGGAGGCCGCACGCACCCGCGCTGCTAACAAAGCCCGAAAGGAAGCTGAGTTGGCTGCTGCCACCGCTGAGCAATCACTAGCATAACCCCTTGGGGCCTCTAAACGGGTCTTGA‑GGGGTTTTGTGCTGAAAGGAGGAACTATATGCGCTCATGCGATATGAACGTTGAGACTGCCGCTGAGATATCAGCTGTGAACGACATGCTGGcaggcgc                                                                                                                                                                     >  1:764239/1‑295 (MQ=255)`  
                                                                                                                                         `gAGTAATGCTGTGGGTGGCCTCAACGGTCGCTGCTAGTCCCGAAGAGGCGAGTGTTACTTCAACAGAAGAAACCTTAACGCCAGCACAGGAGGCCGCACGAACCCGCGCTGCTAACAAAGCCCGAAAGGAAGCTGAGTTGGCTGCTGCCACCGCTGAGCAATCACTAGCATAACCCCTTGGGGCCTCTAAACGGGTCTTGA‑GGGGTTTTTTGCTGAAAGGAGGAACTATATGCGCTCATGCGATATGAACGTTGAGACTGCCGCTGAGTTATCAGCTGTGAACGAcctgtctcttatacac                                                                                                                                                                    >  1:56516/1‑286 (MQ=255)`  
                                                                                                                                         `gAGTAATGCTGGGGGTGGCCTCAACGGTCGCTGCTAGTCCCGAAGAGGAGAGTGTTACTTAAACAGAAGAAACCTTAACGCCAGCACAGGAGGCCGCACGCACCCGCGCTGCTAACAAAGCCCGAAAGGAAGCTGAGTTGGCTGCTGCCACCGCTGAGCAATCACTAGCATAACCCCTTGGGGCCTCTAAACGGGTCTTGA‑GGGGTTTTTTGCTGAAAGGAGGAACTATATGCGCTCATGCGATATGAACGTTGAGACTGCCGCTGAGTTATCAGCTGTGAACGACATTCTGGCGTCTATc                                                                                                                                                                    >  1:121194/1‑301 (MQ=255)`  
                                                                                                                                           `gTAATGCTTGGGGTGGCCTCAACGGTCGCTGCTAGTCCCGAATAGGCGAGTGTTACTTCAACAGAAGAAACCTTAACTCCAGCACAGGAGGCCGCACGCACCCGCGCTTCTAACAAAGCCCGAAAGGAAGCTGAGTTGGCTGCTGCCACCGCTGAGCAATAACTAGCATAACCCCTTGGGGCCTCTAAACGGGTCTTGA‑GGGGTTTTTTGCTGAAAGGAGGAACTATATGCGCTCATGCGATATGAACGTTGAGACTGCCGCTGAGTTATCAGCTGTGAACGACATTCTGGCGTCTATCgg                                                                                                                                                                  >  1:526924/1‑301 (MQ=255)`  
                                                                                                                                           `gTAATGCTGGGGGTGGCCTCAACGGTCGCTGCTAGTCCCGAAGAGGCGAGTGTTACTTCAACAGAAGAAACCTTAACGCCAGCACAGGAGGCCGCACGCACCCGCGCTGCTAACAAAGCCCGAAAGGAAGCTGAGTTGGCTGCTGCCACCGCTGAGCAATCAATAGCATAACCCCTTGGGGCCTCTAAACGGGTCTTGA‑GGGGTTTTTTGCTGAAAGGAGGAACTATATGCGCTCATGCGATATGAACGTTGAGACTGCCGATGAGTTATCAGCTGTGCACGACATTCTGGCGTCTacggg                                                                                                                                                                  >  1:346011/1‑297 (MQ=255)`  
                                                                                                                                           `gTAATGCTGGGGGTGGCCTCAACGGTCGCTGCTAGTCACGAAGAGGCGAGTGTTACTTCAACAGAAGAAACCTTAACGCCAGCACAGGAGGCCGCACGCACCCGCGCTGCTAACAAAGCCCGAAAGGAAGCTGAGTTGGCTGCTGCCACCGCTGAGCAATCACTAGCATAACCCCTTGGGGCCTCTAAACGGGTCTTGA‑GGGGTTTTTTGCTGAAAGGAGGAACTATATGCGCTCATGCGATATGAACGTTGAGACTGCCGCTGAGTTATCAGCTGTGAACGACATTCTGGCGTCTATCgg                                                                                                                                                                  <  1:125685/301‑1 (MQ=255)`  
                                                                                                                                              `aTGCTGGGGGTGGCCTCAACGGTCGCTGCTAGTCCCGAAGAGGCGAGTGTTACTTCAACAGAAGAAACCTTAACGCCAGCACAGGAGGCCGCACGCACCCGCGCTGCTAACAAAGCCCGAAAGGAAGCTGAGTTGGCTGCTGCCACCGCTGAGCAATCACTAGCATAACCCCTTTGGGCCTCTAAACGGGTCTTGA‑GGGGTTTTTTGCTGAAAGGAGGAACTATATGCGCTCATGCGATATGAACGTTGAGACTGCCGCTGAGTTTTCAGCTGTGAACGACATTCTGGCGTCTATCGGTGa                                                                                                                                                               >  1:499749/1‑301 (MQ=255)`  
                                                                                                                                                        `gggCCTCAACGGTCGCTGCTAGTCCCGAAGAGGCGAGTGTTACTTAAACAGAAGAAACCTTAACGCCAGCACAGGAGGCCGCACGCACCCGCGCTGCTAACAAAGCCCGAAAGGAAGCTGAGTTGGCTGCTGCCACCGCTGAGCAATCACTAGCATAACCCCTTGGGGCCTCTAAACGGGTCTTGA‑GGGGTTTTTTGCTGAAAGGAGGAACTATATGCGCTCATGCGATATGAACGTTGAGACTGCCGCTGAGTTATCAGCTGTGAACGACATTCTGGAGTCTATCGGTGAACCTCCGGTa                                                                                                                                                     <  1:289569/300‑1 (MQ=255)`  
                                                                                                                                                        `ggagatgtgtataagagacaggTCCCGAAGAGGCGAGTGTTACTTCAACAGAAGAAACCTTAACGCCAGCACAGGAGGCCGCACGCACCCGCGCTGCTAACAAAGCCCGAAAGGAAGCTGAGTTGGCTGCTGCCACCGCTGAGCAATCACTAGCATAACCCCTTGGGGCCTCTAAACGGGTCTTGA‑GGGGTTTTTTGCTGAAAGGAGGAACTATATGCGCTCATGCGATATGAACGTTGAGACTGCCGCTGAGTTATCAGCTGTGAACGACATTCTGGCGTCTATCGGTGAACCTCCGGTa                                                                                                                                                     <  1:258767/280‑1 (MQ=255)`  
                                                                                                                                                                      `gctgctAGTCCCGACGAGGCAAGTGTTACTTCAACAGTAGAAACCTTATCGCCAGCACAGGAGGCCGCACGCACCCGCGCTGCTAACAAAGCCCGAAAGGAAGCTGAGTTGGCTGCTGCCACCGCTGAGCAATCACTAGCATAACCCCTTGGGGCCTCTAAACGGGTCTTGT‑GGGGTTTTTTGCTGAAAGGAGGAACTATATGCGCTCATGCGATATGAACGTTGAGACTGCCGCTGAGTTATCAGCTGTGAACGACATTCTGGCGTCTATCGGTGAACCTCCGGTATCAACGCTGGAAgg                                                                                                                                       <  1:201272/301‑1 (MQ=255)`  
                                                                                                                                                                       `ctgctAGTCCCGAAGAGGCGAGTGTTTCTTCAACAGAAGAAACCTTAACGCCAGCACAGGAGGCCGCACGCACCCGCGCTGCTAACAAAGCCCGAAAGGAAGCTGAGTTGGCTGCTGCCACCGCTGAGCAATCACTAGCATAACCCCTTGGGGCCTCTAAACGGGTCTTGA‑GGGGTTTTTTGCTGAAAGGAGGAACTATATGCGCTCATGCGATATGAACGTTGAGACTGCCGCTGAGTTATCAGCTGTGAACGACATTCTGGCGTCTATCGGTGAACCTCCGGTATCAACGCTGGAAggg                                                                                                                                      >  1:181914/1‑300 (MQ=255)`  
                                                                                                                                                                         `ccaaGTCCCGAAGCGTCGAGTGTTACTTCAACAGAAGAAACCTGAACGCCAGCACAGGAGGCCGCACGCACCCGCGCTGCTAACAAAGCCCGAAAGGAAGCTGAGTTGGCTGCTGCCACCGCTGAGCAATCTCTAGCATAACCCCTTGGGGCCTCTAAACGGGTCTTGA‑GGGGTTTTTTGCTGAAAGGAGGAACTATATGCGCTCATGCGTTATGAACGTTGAGACTGCCGCTGAGTTATCAGCTGTTAACGACATTCTGGCGTCTATCGGTGAACCTCCTGTATCAACGCTGGAAGGTGa                                                                                                                                    <  1:358460/298‑1 (MQ=255)`  
                                                                                                                                                                             `ctcggagatgtgtataagagacagcAACAGAAGAAACCTTAACGCCAGCACAGGAGGCCGCACGCACCCGCGCTGCTAACAAAGCCCGAAAGGAAGCTGAGTTGGCTGCTGCCACCGATGAGCAATCACTAGCATAACCCCTTGGGGCCTCTAAACGGGTATTGA‑GGGGTTTTTTGCTGAAAGGAGGAACTATATGCGCTCATGCGATATGAACGTTGAGACTGCCGCTGAGTTATCAGCTGTGAACGACATTCTGGCGTCTATCGGTGAACCTCCGGTATCAACGCTGGAAGGTGACGCt                                                                                                                                <  1:645138/277‑1 (MQ=255)`  
                                                                                                                                                                                  `agatgtgtataagagacagcAACAGAAGAAACCTTAACGCCAGCACAGGAGGACGCACGCACCCGCGCTGCTAACAAAGCCCGAAAGGAAGCTGAGTTGGCTGCTGCCACCGCTGAGCAATCACTAGCATAACCCCTTGGGGCCTCTAAACGGGTCTTGA‑GGGGTTTTTTGCTGAAAGGAGGAACTATATGCGCTCATGCGATATGAACGTTGAGACTGCCGCTGAGTTATCAGCTGTGAACGACATTCTGGCGTCTATCGGTGAACCTCCGGTATCAACGCTGGAAGGTGACGCTAACGc                                                                                                                           <  1:729396/282‑1 (MQ=255)`  
                                                                                                                                                                                  `aaagagacaggtgtTACTTCAACAGAAGAAACCTTAACGCCAGCACAGGAGGCCGCACGCACCCGCGCTGCTAACAAAGCCCGAAAGGAAGCTGAGTTGGCTGCTGCCACCGCTGAGCAATCACTAGCATAACCCATTGGGGCATCTAAACGGGTCTTGA‑GGGGTTTTTTGCTGAAAGGAGGAACTATATGCGCTCATGCGATATGAACGTTGAGACTGCCGCTGAGTTATCAGCTGTGAACGACATTCTGGCGTCTATCGGTGAACCTCCGGTATCAACGCTGGAAGGTGACGCTAACGc                                                                                                                           <  1:25053/291‑1 (MQ=255)`  
                                                                                                                                                                                       `ggCGAGTGTTACTTCAACAGAAGAAACCTTAACGCCAGCACAGGAGGCCGCACGCACCCGCTCTGCTAACAAAGCCCGAAAGGAAGCTGAGTTGGCTGCTGCCACCGCTGAGCAATCACTAGCATAACCCCTTGGGGCCTCTAAACGGGTCTTGA‑GGGGTTTTTTGCTGAAAGGAGGAACTATATTCGCTCATGCGATATGAACGTTGAGACTGCCGCTGAGTTATCAGCTGTGAACGACATTCTGGCGTCTATCGGTGAACCTCCGGTATCAACGCTGGAAGGTGACGCTAACGCAGATg                                                                                                                      >  1:684015/1‑301 (MQ=255)`  
                                                                                                                                                                                            `gtgtTACTTCAACAGAAGAAACCTTAACGCCAGCACAGGAGGCCGCACGCACCCGCGCTGCTAACAAAGCCCGAAAGGAAGCTGAGTTGGCTGCTGCCACCGCTGAGCAATCACTAGCATAACCCCTTGGGGCCTCTAAACGGGTCTTGA‑GGGTTTTTTTGCTGAAAGGAGGAACTATATGCGCTCATGCGATATGAACGTTGAGACTGCCGCTGAGTTATCAGCTGTGAACGACATTCTGGCGTCTATCGGTGAACCTCCGGTATCAACGCTGGAAGGTTACGCTAACGCAGATGCAGCg                                                                                                                 >  1:52384/1‑301 (MQ=255)`  
                                                                                                                                                                                            `gtgtTACTTCAACAGAAGAAACCTTAACGCCAGCACAGGAGGCCGCACGCACCCGCGCTGCTAACAAAGCCCGAAAGGAAGCTGAGTTGGCTGCTGCCACCGCTGAGCAATCACTAGCATAACCCCTTGGGGCCTCTAAACGGGTCTTGA‑GGGGTTTTTTGCTGAAAGGAGGAACTATATGCGCTCATGCGATATGAACGTTGAGACTGCCGCTGAGTTATCAGCTGTGAACGACATTCTGGCGTCTATCGGTGAACCTCCGGTATCAACGCTGGAAGGTGACGCTAACGCAGATGCAGCg                                                                                                                 <  1:615705/301‑1 (MQ=255)`  
                                                                                                                                                                                            `gtgtTACTTCAACAGAAGAAACCTTAACGCCAGCACAGGAGGCCGCACGCACCCGCGCTGCTAACAAAGCCCGAAAGGAAGCTGAGTTGGCTGCTGCCACCGCTGAGCAATCACTAGCATAACCCCTTGGGGCCTCTAAACGGGTCTTGA‑GGGGTTTTTTGCTGAAAGGAGGAACTATATGCGCTCATGCGATATGAACGTTGAGACTGCCGCTGAGTTATCAGCTGTGAACGACATTCTGGCGTCTATCGGTGAACCTCCGGTATCAACGCTGGAAGGTGACGCTAACGCAGATGCAGCg                                                                                                                 <  1:42384/301‑1 (MQ=255)`  
                                                                                                                                                                                              `cgtgggctcggagatgtgtataagagacaggCACAGGAGGCCGCACGCACCCGCGCTGCTAACAAAGCCCGAAAGGAAGCTGAGTTGGCTGCTGCCACCGCTGAGCAATCACTAGCATAACCCCTTAGGGCCTCTAAACGGGTCTTGA‑GGGGTTTTTTGCTGAAAGGAGGAACTATATGCGCTCATGCGATATGAACGTTGAGACTGCCGCTGAGTTATCAGCTGTGAACGACATTCTGGCGTCTATCGGTGAACCTCCGGTATCAACGCTGGAAGGTGACGCTAACGCAGATGCAGCGaa                                                                                                               <  1:39537/271‑1 (MQ=255)`  
                                                                                                                                                                                               `gatgtgtataagagacagccTTAACGCCAGCACAGGAGGCCGCACACACCCGCGCTGCTAACAAAGCCCGAAAGGAAGCTGAGTTGGCTGCTGCCACCGCTGAGCAATCACTAGCATAACCCCTTGGGGCCTCTAAACGGGTCTTGA‑GGGGTTTTTTGCTGAAAGGAGGAACTATATGCGCTCATGCGATATGAACGTTGAGACTGCAGCTGAGTTATCAGCTGTGAACGACATTCTGGCGTCTATCGGTGAACCTCCGGTATCAACGCTGGAAGGTGACGCTAACGCAGATGCAGCGAAc                                                                                                              <  1:155278/283‑1 (MQ=255)`  
                                                                                                                                                                                                     `cAACAGAAGAAACCTTAACGCCAGCACAGGAGGCCGCACGCACCCGCGCTGCTAACAAAGCCCGAAAGGAAGCTGAGTTGGCTGCTGCCACCGCTGAGCAATCACTAGCATAACCCCTTGGGGCCTCTAAACGGGTCTTGA‑GGGGTTTTTTGCTGAAAGGAGGAACTATATGCGCTCATGCGATATGAACGTTGAGACTGCCGCTGAGTTATCAGCTGTGAACGACATTCTGGCGTCTATCGGTGAACCTCCGGTATCAACGCTGGAAGGTGACGCTAACGCAGATGCAGCGAACGCTCgg                                                                                                        >  1:652100/1‑301 (MQ=255)`  
                                                                                                                                                                                                       `aCAGAAGAAACCTTAACGCCAGCACAGGAGGCCGCACGCACCCGCGCTGCTAACAAAGCCCGAAAGGAAGCTGAGTTGGCTGCTGCCACCACTGAGCAATAACTAGCATAACACCTTGGGGCCTCTAAACGGGTCTTGA‑GGGGTGTTTTGCTGAAAGGAGGAACTATATGCGCTCATGCGATATGAACGTTGAGACTGCCGCTGAGTTATAAGCTGTGAACGACATTCTGGCGTCTATCGGTGAACCTCCGGTATCAACGCTGGAAGGTGACGCTAACGCAGATGCAGCGAACGCTCGGCg                                                                                                      <  1:25618/301‑1 (MQ=255)`  
                                                                                                                                                                                                         `agaagaAACCTTAACGCCAGCACAGGAGGCCGCACGCACCCGCGCTGCTAACAAAGCCCGAAAGGAAGCTGAGTTGGCTGCTGCCACCGCTGAGCAATCACTAGCATAACCACTTGGGGCCTCTAAACGGGTCTTGA‑GGGGTTTTTTGCTGAAAGGAGGAACTATATGCGCTCATGCGATATGAACGTTGAGACTGCCGCTGAGTTATCAGCTGTGAACGACATTCTGGCGTCTATCGGTGAACCTCCGGTATCAACGCTGGAAGGTGACGCTAACGCAGATGCAGCGAACGCTCGGCGTa                                                                                                    <  1:454936/301‑1 (MQ=255)`  
                                                                                                                                                                                                          `gaagaaACCTTAACGCCAGCACAGGAGTCCGCACGCACCCGCGCTGCTAACAAAGCCCGAAAGGAAGCTGAGTTGGCTGCTGCCACCGCTTAGCAATCACTAGCATAACCCCTTGGGGCCTCTAAACGGGTCTTGA‑GGGGTTTTTTGCTGAAAGGAGGAACTATATGCGCTCATGCGATATGAACGTTTAGACTGCCGCTGAGTTATCAGCTGTGAACGACATTCTGGCGTCTATCGGTGAACCTCCGGTATCAACGCTGGAAGGTGACGCTAACGCAGATGCAGCGAACGCTCGGCGTAt                                                                                                   >  1:766055/1‑301 (MQ=255)`  
                                                                                                                                                                                                          `gaagaaACCTTAACGCCAGCACAGGAGGCCGCACGCACCCGCGCTGCTAACAAAGCCCGAAAGGAAGCTGAGTTGGCTGCTGCCACCGATGAGCAATCACTAGCATAACCCCTTGGGGCCTCTAAACGGGTCTTGA‑GGGGTTTTTTGCTGAAAGGAGGAACTATATGCGCTCATGCGATATGAACGTTGAGACTGCCGCTGAGTTATCAGCTGTGAACGACATTCTGGCGTCTATCGGTGAACCTCCGGTATCAACGCTGGAAGGTGACGCTAACGCAGATGCAGCGAACGCTCGGCGTAt                                                                                                   >  1:169141/1‑301 (MQ=255)`  
                                                                                                                                                                                                                      `aCGCCAGCACAGGAGGCCGCACGCACCCGCGCTGCTAACAAAGCCCGAAAGGAAGCTGAGTTGGCTGCTGCCACCGCTGAGCAATCACTAGCATAACCCCTTGGGGCCTCTAAACGGGTCTTGA‑GGGGTTTTTTGCTGAAAGGAGGAACTATATGCGCTCATGCGATATGAACGTTGAGACTGCCGCTGAGTTATCAGCTGTGAACGACATTCTGGCGTCTATCGGTGAACCTCCGGTATCAACGCTGGAAGGTGACGCTAACGCAGATGCAGCGAACGCTCGGCGTATTCTCAACAAGAt                                                                                       <  1:437206/301‑1 (MQ=255)`  
                                                                                                                                                                                                                           `aGCACAGGAGGCCGCACGCACCCGCGCTGCTAACAAAGCCCGAAAGGAAGCTGAGTTGGCTGCTGCCACCGCTGAGCAATCACTAGCATAACCCCTTGGGGCCTCTAAACGGGTCTTGA‑GTGGTTTTTTGCTGAAAGGAGGAACTATATGCGCTCATGCGATATGAACGTTGAGACTGCCGCTGAGTTATCAGCTGTGAACGACATTCTGGCGTCTATCGGTGAACCTCCGGTATCAACGCTGGAAGGTGACGCTAACGCAGATGCAGCGAACGCTCGGCGTATTCTCAACAAGATTAAcc                                                                                  <  1:665465/301‑1 (MQ=255)`  
                                                                                                                                                                                                                            `gCACAGGAGGCCGCACGCACCCGCGCTGCTAACAAAGCCCGAAAGGAAGCTGAGTTGGCTGCTGCCACCGCTGAGCAATAACTAGCATAACCCCTTGGGGCCTCTAAACGGGTCTTGA‑GGGGTTTTTTGCTGAAAGGAGGAACTATATGCGCTCATGCGATATGAACGTTGAGACTGCCGCTGAGTTATCAGCTGTGAACGACATTCTTGCGTCTATCGGTGAACCTCCGGTATCAACGCTGGAAGGTGACGCTAACGCCGATGCAGCGAACGCTCGGGGTATTCTCAACAAGATTTAccc                                                                                 >  1:492489/1‑300 (MQ=255)`  
                                                                                                                                                                                                                            `gCACAGGAGGCCGCACGCACCCGCGCTGCTAACAAAGCCCGAAAGGAAGCTGAGTTGGCTGCTGCCACCGCTGAGCAATAACTAGCATAACCCCTTGGGGCCTCTAAACGGGTCTTGA‑GGGGTTTTTTGCTGAAAGGAGGAACTATATGCGCTCATGCGATATGAACGTTGAGACTGCCGCTGAGTTATCAGCTGTGAACGACATTCTGGCGTCTATCGGTGAACCTCCGGTATCAACGCTGGAATGTGACGCTAACGCAGATGCAGCGAACGCTCGGCGTATtcctgtctcttatacaca                                                                                 >  1:269896/1‑285 (MQ=255)`  
                                                                                                                                                                                                                              `acaGGAGGCCGCATGCACCCGCGCTGCTAACAAAGCCCGAAAGGAAGCTCAGTTGGCTGCTGCCACCGCTGAGCAATCACTAGCATAACCCCTTGGGGCCTCTAAACGGGTCTTGA‑GGGGTTTTTTGCTGAAAGGAGAAACTATATGCGCTCATGCGATATGAACGTTGAGACTGCCGCTGAGTTATCAGCTGTGAACGACATTCTGGCGTCTATCGGTGAACCTCCGGTATCAACGCTGGAAGGTGACGCTAACGCAGATGCAGCGAACGCTCGGCGTATTCTCAACAAGATTAACCGAc                                                                               >  1:333004/1‑301 (MQ=255)`  
                                                                                                                                                                                                                               `caGGAGGCCGCACGCACCCGCGCTGCTAACAAAGCCCGAAAGGAAGCTGAGTTGGCTGCTGCCACCGCTGAGCAATAACTAGCATAACCCCTTTGGGCCTCTAAACTGGTCTTGA‑GGGGTTTTTTGCTGAAAGGAGGAACTATATGCGCTCATGCGATATGAACGTTGAGACTGCCGCTGAGTTATCAGCTGTGAACGACATTCTGGCGTCTATTGGTGAACCTCCGGTATCAACGCTGGAAGGTGACGCTAACGCAGATGCAGCGAACGCTCGGCGTATTCTgaacctgtctcttataca                                                                              >  1:754428/1‑287 (MQ=255)`  
                                                                                                                                                                                                                                         `cgtcagatgtgtataagagacaggCCCGAAAGGAAGCTGAGTTGGCTGCTGCCACCGCTTAGCAATCACTAGCATAACCCCTTGGGGCCTCTAAACGGGTCTTGA‑GGGGTTTTTTGCTGAAAGGAGGAACTATATGCGCTCATGCGATATGAACGTTGAGACTGCCGCTGAGTTATCAGCTGTGAACGACATTCTGGCGTCTATCGGTGAACCTCCGGTATCAACGCTGGAAGGTGACGCTAACGCAGATGCAGCGAACGCTCGGCGTATTCTCAACAAGATTAACCGACAGCTTCAAtct                                                                    >  1:339872/24‑301 (MQ=255)`  
                                                                                                                                                                                                                                             `cacCCGCGCTGCTAACAAAGCCCGAAAGGAAGCTGAGTTGGCTGCTGCCACCGCTGAGCAATCACTAGCATAACCCCTTGTGGCCTCTAAACGGGTCTTGA‑GGGGTTTTTTGCTGAAAGGAGGAACTATATGCGCTCATGCGATATGAACGTTGAGACTGCCGCTGAGTTATCAGCTGTGAACGACATTCTGGCGTCTATCGGTGAACCTCCGGTATCAACGCTGGAAGGTGACGCTAACGCAGATGCAGCGAACGCTCGGCGTATTCCCAACAAGaactgtctcttatacacatctccga                                                                >  1:415248/1‑277 (MQ=255)`  
                                                                                                                                                                                                                                             `cacCCGCGCTGCTAACAAAGCCCGAAAGGAAGCTGAGTTGGCTGCTGCCACCGCTGAGCAATCACTAGCATAACCCCTTGGGGCCTCTAAACGGGTCTTGA‑GGGGTTTTTTGCTGAAAGGAGGAACTATATGCGCTCATGCGATATGAACGTTGAGACTGCCGCTGAGTTATCAGCTGTGAACGACATTCTGGCGTCTATCGGTGAACCTCCGGTATCAACGCTGGGAGGTGACGCTAACGCAGATGCAGCGAACGCTCGGCGTATTCTCAACAAGATTAACCGACAGATTCAATCTCGtg                                                                >  1:549138/1‑301 (MQ=255)`  
                                                                                                                                                                                                                                                 `cgcgCTGCTAACAAAGCCCGAAAGGAAGCTGAGTTGGCTGCTGCCACCGCTGAGCAATAACTAGCATATCCCCTTGGGGCCTCTAAACGGGTCTTGA‑GGGGTTTTTTGCTGAAAGGAGGAACTATATGCGCTAATGCAATATGAACGTTGAGACTGCAGCTGAGTTATCAGCTGTGAACGACATTCTGGCGTCTATCGGTGATCCTCCGGCATCAACGCTAGAAGGTGACGCTAACGCAGATGCAGCGAACGCTCGGCGTATTCTCAACAAGATTAACCGACAGATTCAATCTCGtggatg                                                            <  1:47132/301‑1 (MQ=255)`  
                                                                                                                                                                                                                                                     `ctgctAACAAAGCCCGAAAGGAAGCTGAGTTGGCTGCTGCCACCGCTGAGCAATCACTAGCATAACCCCTTGGGGCCTCTAAACGGGTCTTGA‑GGGGTTTTTTGCTGAAAGGAGGAACTATATGCGCTCATGCGATATGAACGTTGAGACTGCCGCTGAGTTATCAGCTGTGAACGACATTCTGGCGTCTATCGGTGAACCTCCGGTATCAACGCTGGAAGGTGACGCTAACGCAGATGCAGCGAACGCTCTGCGTATTCTCAACAAGATTAACCGACAGATTCAATCTCGTGGATGGACg                                                        <  1:92042/301‑1 (MQ=255)`  
                                                                                                                                                                                                                                                             `gatgtgtataagagacaggAGTTGGCTGCTGCCACCGCTGAGCAATAACTAGCATAACCCCTTGGGGCCTCTAAACGGGTCTTGA‑GGGGTTTTTTGCTGAAACGAGGAACTATATGCGCTCATACGATATGAACGTTGAGACTGCCGCTGAGTTATCAGCTGCGAACGACATTCTGGCGTCTATCGGTGAACCTCCGGTATCAACGCTGGAAGGTGACGCTAACGCAGATGCAGCGAACGCTCGGCGTATTCTCAACAAGATTAACCGACAGATTCAATCTCGTGGATGGACGTTCAACAt                                                <  1:347820/283‑1 (MQ=255)`  
                                                                                                                                                                                                                                                                  `cggagatgtgtataagagacaggctgcCACCGCTGAGCAATCACTAGCATAACCCCTTGGGGCCTCTAAACGGGTCTTGA‑GGGGTTTTTTGCTGAAATGAGGAACTATATGCGCTCATGCGATATGAACGTTGAGACTGCCGCTGAGTTATCAGCTGTGAACGACATTCTGGCGTCTATCGGTGAACCTCCGGTATCAACGCTGGAAGGTGACGCTAACGCAGATGCAGCGAACGCTCGGCGTATTCTCAACAAGATTAACCGACAGATTCAATCTCGTGGATGGACGTTCAACATTGAgg                                           <  1:653232/279‑1 (MQ=255)`  
                                                                                                                                                                                                                                                                    `gAAAGGAAGCTGAGTTGGCTGCTGCCACCGCTGAGCAATCACTAGCATAACCCCTTGGGGCCTCTAAACGGGTCTTGAGGGGGTTTTTTGCTGAAAGGAGGAACTATATGCGCTCATGCGATATGAACGTTGAGACTGCCGCTGAGTTATCAGCTGTGAACGACATTCTGGCGTCTATCGGTGAACCTCCGGTATCAACGCTGGAAGGTGACGCTAACGCAGATGCAGCGAACGCTCGGCGTATTCTCAACAAGATTAACCGACAGATTCAATCTCGTGGATGGACGTTCAACATCGAGGa                                          <  1:459770/301‑1 (MQ=255)`  
                                                                                                                                                                                                                                                                    `gAAAGGAAGCTGAGTTGGCTGCTGCCACCGCTGAGCAATCACTAGCATAACCCCTTGGGGCCTCTAAACGGGTCTTGA‑GGGGTTTTTTTCTGAAAGGAGGAACTATATGCGCTCATGCGATATGAACGTTGAGACTGCCGCTGAGTTATCAGCTGTGAACGACATTCTGGCGTATATCGGTGAACCTACGGTATCAACGCTGGAAGGTGACGCTAACGCAGATGCAGCGAACGCTCGGCGTATTCTCAACAAGATTAACCGACAGATTCAATCTCGTGGATGGACGTTCAACATTGAgtca                                         >  1:383076/1‑298 (MQ=255)`  
                                                                                                                                                                                                                                                                     `aaaGGAAGCTGAGTTGGCTGATGCCACCGCTGAGCAATCACTAGCATAACCCCTTGGGGCCTCTAAACGGGTCTTGA‑GGGGTTTTTTGCTGAAAGGAGGAACTATATGCGCTCATGCGATATGAACGGTGAGACTGCCGCTGAGTTATCAGCTGTGAACGACATTCTGGCGTCTATCGGTGAACCTCCGGTATCAACGCTGGAAGGTGACGCTAACGCAGATGCAGCGAACGCTCGGCGTATTCTCAACAAGATTAACCGACAGATTCAATCTCGTAGATGGACGTTCAACCTTGAggcgg                                        >  1:103493/1‑298 (MQ=255)`  
                                                                                                                                                                                                                                                                         `gAAGCTGAGTTGGCTGCTGCCACCGCTGAGCAATCACTAGCATAACCCCTTGTGGCCTCTAAACGGGTCTTGA‑GGGGTTTTTTGCTGAAAGGAGGAACTATATGCGCTCATGCGATATGAACGTTGAGACTGCCGCTGAGTTATCAGCTGTGAACGACATTCTGGCGTCTATCGGTGAACCTCCGGTATCAACGCTGGAAGGTGAAGCTAACGCAGATGCAGCGAACGCTCGGCGTATTCTCAACAAGATTAACCGACAGATGCAATCTCGTGGATGGACGTTCAACATTGAGGAAGGCaa                                    >  1:108989/1‑300 (MQ=255)`  
                                                                                                                                                                                                                                                                            `ggctcggagatgtgtataagagacaggCAATAACTAGCATAACACCTTGGGGCCTCTAAACGGGTCTTGA‑GGGGTTTTTTGCTGAAAGGAGGAACTATATGCGCTCATGCGATATGAACGTTGAGACTGCCGCTGAGTTATCAGCTGTGAACGACATTCTGGCGTCTATCGGTGAACCTCCGGTATCAACGCTGGAAGGTGACGCTAACGCAGATGCAGCGAACGCTCGGCGTATTCTCAACAAGATTAACCGACAGATTAAATCTCGTGGATGGACGTTCAACATTGAGGAAGGCATAAc                                 <  1:540874/275‑1 (MQ=255)`  
                                                                                                                                                                                                                                                                             `cTGAGTTGGCTGCTGCCACCGCTGAGCAATCACTAGCATAACCCCTTGGGGCCTCTAAACGGGTCTTGA‑GGGGTTTTTTGCTGAAAGTAGGAACTATATGCGCTTCTGCGATATGAACGTTGAGACTGCCGCTGAGTTATTAGCTGTGAACGACATTCTGGCGTCTATCGGTGAACCTCCGGTATCAACGCTGGTAGGTGACGCTAACGCATATGCAGCGAACGCTCGGCGTATTCCCACCAAGATTAACTGACAGATTCAATCTCGTGGATGGACGTTCAACATTGAGGAAGGCATAACg                                >  1:51975/1‑301 (MQ=255)`  
                                                                                                                                                                                                                                                                               `gAGTTGGCTGCTGCCACCGCTGAGCAATCACTAGTATAACCCCTTGGGGCCTCTAAACGGGTCTTGA‑GGGGTTTTTTGCTGAAAGGAGGAACTATATGCGCTCATACGATATGAACGTTGAGACTGCCGCTGAGTTATCAGCTGTGAACGACATTCTGGCGTCTATCGGTGAACCTCCGGTATCAACGCTGGAAGGTGACGCTAACGCAGATGCAGCGCACGCTCGGCGTATTCTCAACAAGATTAAACGACATATTCAATCTCGTGGATGGACGATCAACAtctgtctcttatacacatc                              >  1:673248/1‑283 (MQ=255)`  
                                                                                                                                                                                                                                                                                 `ttgggCTGCTGCCACCGCTGAGCAATCACTATCATAACACCTTGGGGCCTCTAAACGGGTCTTGA‑GGGGTTTTTTGCTGAAAGGAGGAACTATATGCGCTCATTCGATATGAACGTTGAGACTGCCGCTGAGTTATCAGCTGTGAACGACATTCTGGCGTCTATCGGTGAACCTCCGGTATCAACGCTGGAAGGTGACGCTAACGCAGATGCAGCGAACGCTCGGCGTATTCTAAACAAGATTAACCGACAGATTCAATCTCGTGGATGGACGTTCAACATTGAGGAAGGCATAACGctac                            <  1:694221/298‑1 (MQ=255)`  
                                                                                                                                                                                                                                                                                 `gttggttgCTGCCACCGCTGAGCAATCACTAGCATAACCCCTTGGGGCCTATAAACGGGTCTTGA‑GGGGTTTTTTGCTGAAAGGAGGAACTATATGCGCTCATGCGATATGAACGTTGAGACTGCCGCTGAGTTATCAGCTGTGAATGACATTCTGGCGTCTATCGGTGAACCTCCGGTATCAACGCTGGAAGGTGACGCTAACGCAGATGCAGCGAACGCTCGGCGTATTCTCAACAAGATTAACCGACAGATTCAATCTCGTGGATGGACGTTCAACATTGATGAAGGCATAACGctac                            <  1:800972/301‑1 (MQ=255)`  
                                                                                                                                                                                                                                                                                 `gTTGGCTGCTGCCACCGCTGAGCAATCACTAGCATAACCCCTTGGGGCCTCTAAACGGGTCTTGA‑GGGGTTTTTTGCTGAAAGGAGGAACTATATGCGCTCATGCGATATGAACGTTGAGACTGCCGCTGAGTTATCAGCTGTGAACGACATTCTGGCGTCTATCGGTGAACCTCCGGTATCAACGCTGGAAGGTGACGCTAACGCAGATGCAGCGAACGCTCGGCGTATTCTCAACAAGATTAACCGACAGATTCAATCTCGTTGATGGACGTTCAACATTGAGGAAGGCATAACGctac                            >  1:639285/1‑301 (MQ=255)`  
                                                                                                                                                                                                                                                                                 `gTTGGCTGCTGCCACCGCTGAGCAATAACTAGCATAACCCCTTGGGGCCTCTAAACGGGTCTTGA‑GGGGTTTTTTGCTGAAAGGAGGAACTATATGCGCTCATGCGATATGAACGTTGAGACTGCCGCTGAGTTATCAGCTGTGAACGACATTCTGGCGTCTATCGGTGAACCTCCGGTATCAACGCTGGAAGGTGACGCTAACGCAGATGCAGCGAACGCTAGGCGTATTCTCAACAAGATTAACCGACAGATTCAATCTCGTGGATGGACGTTCAACATTGAGGAAGGCATAACGctac                            <  1:657044/301‑1 (MQ=255)`  
                                                                                                                                                                                                                                                                                 `gTTGGCTGCTGCAACCGCTGAGCAATCACTAGCATAACCCCTTGGGGCCTCTAAACGGGTCTTTA‑GGGGTTTTTTGCTGAAAGGAGGAACTATATGCGCTCATGCGATATGAACGTTGAGACTGCCGATGAGTTATCAGCTGTGAACGACATTCTGGCGTCTATCGGTGAACCTCCGGTATCAACGCTGGAAGGTGACGCTAACGCAGATGCAGCGAACGCTCGGCGTATTTTCAACAAGATTAACCGACAGATTCAATCTCGTGGATGGACGTTCAACATTGAGGAAGGCATAACGctac                            <  1:755396/301‑1 (MQ=255)`  
                                                                                                                                                                                                                                                                                 `cagggCTGCTGCCACCGCTGAGCAATAACTAGCATAACCCCTTGGGGCCTCTAAACGTGTCTTGA‑GGGGTTTTTTGCTGAAAGGAGGAACTATATGCGCTCATGCGATATGAACGTTGAGACTGCCGCTGAGTTATCAGCTGTGAACGACATTCTGGCGTCTATCGGTGAACCTCCGGTATCAACGCTGGAAGGTGACGCTAACGCAGATGCAGCGAACGCTCGGCGTATTCTCAACAAGATTAACCGACAGATTCAATCTCGTGGATGGACGTTCAACATTGAGGAAGGCATAACGctac                            <  1:211908/298‑1 (MQ=255)`  
                                                                                                                                                                                                                                                                                   `tGGCTGCTGCCCCCGCTGAGCAATCACTAGCATAACCCCCTGGGGCCTCTAAACGGGTCTTGA‑GTGGTTTTTTGCTGAAAGGAGGAACTATATGCGCTCATGCGATATGAACGTTGATACTGCCGCTGAGTTATCAGCTGTGAACGACATTCTGGCGTCTATCGGTGAACCTCCGGTATCAACGCTGGAAGGTGACGCTAACGTAGATGCAGCGAACGCTCGGCGTATTCTCAACAAGATTAACCGACAGATTCAATCTCGTGGATGGACGTTCAACATTGAGGAAGGCATAACGctacta                          <  1:809908/301‑1 (MQ=255)`  
                                                                                                                                                                                                                                                                                          `cgcCACCGCTGAGCAATCACTAGCATAACCCCTTGGGGCCTCTAAACGGGTCTTGA‑GGGGTTTTTTGCTGAAAGGAGGAACTATATGCGCTCATGCGATATGAACGTTGAGACTGCCGCTGAGTTATCAGCTGTGAACGACATTCTGGCGTCTATCGGTGAACCTCCGGTATCAACGCTGGAAGGTGACGCTAACGCAGATGCAGCGAACGCTCGGCGTATTCTCAACAAGATTAACCGACAGATTCAATCTCGTGGATGGACGTTCAACATTGAGGAAGGCATAACGCTACTAACTGATg                   <  1:302096/300‑1 (MQ=255)`  
                                                                                                                                                                                                                                                                                               `ccGCTGAGCAATCACTAGCATAACCCCTTGGGGCCTCTAAACGGGTCTTGA‑GGGGTTTTTTGCTGAAAGGAGGAACTATATGCGCTCATGCGATATGAACGTTGAGACTGCCGCTGAGTTATCAGCTGTGAACTACATTCTGGCGTCTATCGGTGAACCTCCGGTATCAACGCTGGAAGGTGACGCTAACGCAGATGCATCGAACGCTCGGCGTATTCTCAACAAGATTAACCGACAGATTCAATCTCGTGGATGGACGTTCAACATTGAGGAAGGCATAACGCTACTACCTGATGTTTAc              >  1:46811/1‑301 (MQ=255)`  
                                                                                                                                                                                                                                                                                               `ccGCTGAGCAATCACTAGCATAACCCCTTGGGGCCTCTAAACGGGACTTGA‑GGGGTTTTTTGCTGAAAGGAGGAACTATATGCGCTCATGCGATATGAACGTTGAGACTGCCGCTGAGTTATCAGCTGTGAACGACATTCTGGCGTCTATCGGTTAACCTCCGGTATCAACGCTGGAAGGTGACTCTAATGCAGATGCAGCGAACGCTCGGCGTATTCTCAACAAGATTAACCGACAGATTCAATCTCGTGGATGGACGTTCAACATTGAGGAAGGAATAACGcctgtctcttatacacct              >  1:447671/1‑284 (MQ=255)`  
                                                                                                                                                                                                                                                                                               `ccGCTGACCAATCACTAGCATAACCCCTTGGGGCCTCTAAACGGGTCTTGA‑GGGGTTTTTTGCTGAAATGAGGAACTATATGCGCTCATGCGATATGAACGTTGAGACTGCCGCTGAGTTATCAGCTGTGAACGACATTCTGGCGTCTATCGGTGAACCTCCGGTATCAACGCTGGAAGGTTACGCTAACGCAGATGCAGCGAACGCTCGGCGTATTCTCAACAAGATTAACCGACAGATTCAATCTCGTGGATGGACGTTCAACATTGAGGAAGGCATAACGCTACTACCTGATGTTTAc              >  1:246017/1‑301 (MQ=255)`  
                                                                                                                                                                                                                                                                                                  `cTGAGCAATAACTAGCATAACCCCTTGGGGCCTCTAAACGGGTCTTGA‑GGGGTTTTTTGCTGAAAGGAGGAACTATATGCGCTCATGCGATATGAACGTTGAGACTGCCGCTGAGTTATCAGCTGTGAACGACATTCTGGCGTCTATCGGTGAACCTCCGGTATCAACGCTGGAAGGTGACGCTAACGCAGATGCAGCGAACGCTCGGCGTATTCTCAACAAGATTAACCGACAGATTCAATCTCGTGGATGGACGTTCAACATTGAGGAAGGCATAACGCTACTACCTGATGTTTACTcc           <  1:428624/301‑1 (MQ=255)`  
                                                                                                                                                                                                                                                                                                      `gCAATCACTAGCATAACCCCTTGGGGCCTCTAAACGGGTCTTGA‑GGGGTTTTTTGCTGAAAGGAGGAACTATATGCGCTCATGCGATATGAACGTTGAGACTGCCGCTGAGTTATCAGCTGTGAACGACATTCTGGCGTCTATCGGTGAACCTCCGGTATCAACGCTGGAAGGTGACGCTAACGCAGATGCAGCGAAAGCTCGGCGTATTCTCAACAAGATTAACCGACAGATTCAATCTCGTGGATGGACGTTCAACATTGAGGAAGGCATAACGCTACTACCTGATGTTTACTCCAAcc       <  1:641304/301‑1 (MQ=255)`  
                                                                                                                                                                                                                                                                                                          `tAACTAGCATAACCCCTTGGGGCCTCTAAACGGGTCTTGA‑GGGGTTTTTTGCTGAAAGGAGGAACTATATGCGCTCATGCGATATGAACGTTGAGACTGCCGCTGAGTTATCAGCTGTGAACGCCATTCTGGCGTCTATCGGTGAACCTCCGGTATCAACGCTGGAAGGTGACGCTAACGCAGATGCAGCGAACGCTCGGCGTATTCTCAACAAGATTAACCGACAGATTCAATCTAGTGGATGGACGTTCAACATTGAGGAAGGCATAACGCTACTACCTGATGTTTACTCCAACCTGAt   >  1:583329/1‑301 (MQ=255)`  
                                                                                                                                                                                                                                                                                                          `tAACTAGCATAACCCCTTGGGGCCTCTAAACGGGTCTTGA‑GGGGTTTTTTGCTGAAAGGAGGAACTATATGCGCTCATGCGATATGAACGTTGAGACTGCCGCTGAGTTATCAGCTGTGAACGACATTCTGGCGTCTATCGGTGAACCTCCGGTATCAACGCTGGAAGGTGACGCTAACGCAGATGCAGCGAACGCTCGGCGTATTCTAAACAAGATTAACCGACAGATTCAATCTAGTGGATGGACGTTCAACATTGAGGAAGGCATAACGcctgtctcttatacacatctccgagcccc   >  1:505809/1‑273 (MQ=255)`  

                                                                                                                                                                                                                                                                                                           `|`                                                                                                                                                                                                                                                                                                               
`GTACTGTTAAGCTGCGTGACTTGGCTCTGGAGCGCGCTCGCCGTGCTAACTTCCAAGCGGACCAGATTATCGCTAAGTACGCAATGGGCCACGGTGGTCTTCGCCCAGAAGCTGCTGGTGCAGTGGTTTTCAAAGTGGAGTAATGCTGGGGGTGGCCTCAACGGTCGCTGCTAGTCCCGAAGAGGCGAGTGTTACTTCAACAGAAGAAACCTTAACGCCAGCACAGGAGGCCGCACGCACCCGCGCTGCTAACAAAGCCCGAAAGGAAGCTGAGTTGGCTGCTGCCACCGCTGAGCAATAACTAGCATAACCCCTTGGGGCCTCTAAACGGGTCTTGA‑GGGGTTTTTTGCTGAAAGGAGGAACTATATGCGCTCATACGATATGAACGTTGAGACTGCCGCTGAGTTATCAGCTGTGAACGACATTCTGGCGTCTATCGGTGAACCTCCGGTATCAACGCTGGAAGGTGACGCTAACGCAGATGCAGCGAACGCTCGGCGTATTCTCAACAAGATTAACCGACAGATTCAATCTCGTGGATGGACGTTCAACATTGAGGAAGGCATAACGCTACTACCTGATGTTTACTCCAACCTGATT  >  NC_001604.1/23862‑24461`

  

Alignment Legend

Aligned base mismatch/match (shaded by quality score): `ATCG/ATCG` `< 3 ≤` `ATCG/ATCG` `< 12 ≤` `ATCG/ATCG` `< 25 ≤` `ATCG/ATCG` `< 36 ≤` `ATCG/ATCG` `< 38 ≤` `ATCG/ATCG`

Unaligned base: `atcg`    Masked matching base: `atcg`    Alignment gap: `‑`    Deleted base: `‑`
